# Advanced methods in deuterium metabolic imaging

**DOI:** 10.1007/s10334-026-01322-1

**Published:** 2026-01-24

**Authors:** Fabian Niess, Bernhard Strasser, Bernard Lanz, Wolfgang Bogner

**Affiliations:** 1 High-Field MR Center, Department of Biomedical Imaging and Image-Guided Therapy, Medical University of Vienna, Lazarettgasse 14, 1090 Vienna, Austria; 2 CIBM Center for Biomedical Imaging, Lausanne, Switzerland; 3 CIBM Pre-Clinical Imaging EPFL, Metabolic Imaging Section, École polytechnique fédérale de Lausanne (EPFL), Lausanne, Switzerland; 4 Christian Doppler Laboratory for MR Imaging Biomarkers (BIOMAK), Vienna, Austria; 5 Comprehensive Center for AI in Medicine (CAIM), Medical University of Vienna, Vienna, Austria

**Keywords:** Deuterium metabolic imaging, Multinuclear MR spectroscopy, Metabolic mapping, Kinetic modeling, Deuterium-labeled tracers

## Abstract

**Background:**

Deuterium metabolic imaging (DMI) has recently been established as a versatile MR-based technique for in vivo mapping of glucose and other metabolic pathways using safe, non-ionizing ^2^H-labeled tracers.

**Objective:**

In this review, methodological advances in DMI over the past decade are summarized, spanning hardware, acquisition, reconstruction, and quantification.

**Approach and Outline:**

Developments in multinuclear system modifications and dual-tuned head and body coils that enable 3D DMI at clinical and ultra-high field strengths are outlined. Efficient spatial–spectral encoding strategies and balanced steady-state-free-precession-based MRSI, which improve SNR efficiency and spatiotemporal resolution, are reviewed together with temporally interleaved ^1^H/^2^H acquisitions that integrate DMI into standard MRI workflows. Indirect ^1^H-observed deuterium detection (QELT) is described as a complementary approach for sites without multinuclear hardware. On the reconstruction side, model-based, low-rank and AI-driven methods are highlighted for de-noising, accelerated sampling, and robust spectral–temporal fitting.

**Outlook:**

Current strategies for concentration estimation, kinetic modeling, and treatment of label loss are discussed, positioning DMI as a promising complement to FDG-PET and ^13^C-MRS for studying metabolism in neurological, oncological and systemic disease.

## Introduction

Over the past decades, metabolic imaging has established a central role in clinical research by elucidating underlying biochemical processes in health and disease and adding valuable complementary information to standard anatomical imaging [1]. Especially positron emission tomography (PET) has been widely adopted in clinical oncological and neurological routine to map tissue uptake of radioactive labeled tracers, providing high sensitivity and excellent quantitative accuracy [2, 3]. However, PET imaging inherently provides only limited (indirect) insights into downstream metabolism due to trapping of the tracer in the early metabolic steps and the ionizing nature of applied tracers restricts frequent use in longitudinal follow-up studies and pediatric imaging.

Magnetic resonance spectroscopy (MRS) and magnetic resonance spectroscopic imaging (MRSI) offer complementary non-invasive information on metabolic profiles in health and disease [4, 5]. While functional studies have been demonstrated, proton (^1^H)-based MRS and MRSI are most commonly used to observe steady-state metabolite levels. Similar to fluorodeoxyglucose (FDG)-PET, non-proton MR techniques, such as ^13^C-MRS enable estimation of metabolic turnover rates of glucose via time-resolved detection of downstream metabolites, i.e., glutamate, glutamine, or lactate, e.g., in brain studies [6]. Despite their potential, widespread clinical translation of these techniques remains limited. One reason is the low sensitivity due to the low gyromagnetic ratio and long *T*_1_ relaxation. Another practical limitation is the very large ^13^C chemical shift range, which can complicate broadband excitation/localization and decoupling, ultimately compromising the optimal acquisition of widely separated resonances within a single scan, such as ^13^C-glucose and ^13^C-glutamate/glutamine.

Ultimately, this often restricts thermally polarized ^13^C tracer studies of low-concentration downstream metabolites to large single voxel or surface coil localization [6]. However, spatially resolved ^13^C measurements can also be achieved for high-concentration compounds, such as glycogen [7] and lipids [8], and via indirect proton-observed ^1^H-[^13^C] detection strategies [9, 10].

Hyperpolarized (HP) ^13^C MRSI, most commonly based on dissolution DNP of [1-^13^C] pyruvate, has become the most widely adopted approach for dynamic ^13^C metabolic imaging studies in humans by overcoming the intrinsically low sensitivity of thermally polarized ^13^C. Key clinical milestones include the first in-human demonstration in prostate cancer [11], establishing feasibility and safety, followed by translation to cardiac metabolic imaging [12] and studies of human brain metabolism [13–15]. The major strengths of HP-^13^C MRSI are its very high instantaneous SNR and the ability to capture fast enzymatic conversions (e.g., pyruvate to lactate/bicarbonate) within clinically practical time scales, whereas limitations include the need for dedicated polarization infrastructure, time critical on-site tracer preparation and the finite lifetime of the hyperpolarized signal (few min after injection).

Deuterium metabolic Imaging (DMI) has emerged as another promising alternative MR-based technique to dynamically image functional and metabolic processes after administration of safe deuterium (^2^H or D)-labeled tracers, such as ^2^H-glucose [16–18], ^2^H_9_-choline [19, 20], beta-hydroxybutyrate ^2^H_4_-BHB [21] or ^2^H_2_O [22–24]. DMI detects the NMR resonance of ^2^H, a stable isotope of hydrogen featuring a 6.5-fold lower gyromagnetic ratio with a natural abundance of 0.015%. This leads to an inherently low SNR, but also reduced background signals, and therefore, sparse frequency spectra typically including single labeled water (DHO, 4.8 ppm) and lipid (1.4 ppm) resonances with similar chemical shifts (in ppm) compared to conventional ^1^H spectra [25]. Additionally, natural abundance resonances have concentrations that are similar compared to administered ^2^H-labeled tracers, e.g., glucose (Glc 3.9 ppm), and downstream metabolites such as combined glutamate + glutamine (glx, 2.4 ppm) or lactate (1.4 ppm). This is a major advantage of DMI applications as it eliminates the need for water or lipid suppression, while water signals can serve as internal reference for concentration estimation. However, reliable quantification of lactate would severely benefit from sophisticated lipid removal strategies [26].

Despite its low sensitivity, DMI benefits from the favorable relaxation properties of ^2^H, i.e., dominating quadrupolar relaxation effects leading to fast longitudinal and transversal relaxation (i.e., short *T*_1_ and *T*_2_ relaxation constants), which enables short repetition times and rapid signal averaging for MRS and MRSI acquisitions under fully relaxation conditions to increase SNR substantially [27]. Nonetheless, the modest sensitivity of ^2^H requires either larger voxel sizes, longer acquisition times, or higher magnetic field strengths to achieve clinically meaningful spatial resolution.

Similar to FDG-PET, DMI enables the dynamic assessment of 3D-resolved glucose uptake, while simultaneously assessing downstream metabolism to estimate metabolic fluxes and calculate turnover rates comparable to ^13^C-MRS, but with superior spatial resolution. Compared to HP ^13^C MRSI, DMI offers a simpler and more scalable workflow without a polarizer, supports longer observation windows and repeated measurements, but with lower sensitivity. With respect to spatial resolution, DMI positions itself between ^13^C-MRS, HP ^13^C MRSI, and FDG-PET and offers a unique combination of safety, simplicity and spectral specificity with clinically attractive spatial and temporal resolution. However, further improvements in low SNR are warranted to improve spatial resolution and reduce scan times of dynamic DMI sessions that still limited its potential use in clinical routine. Beyond labeled- tracer approaches, there is growing interest in proton-based glucose MRI, most prominently glucoCEST and dynamic glucose enhancement (DGE). GlucoCEST was introduced to detect glucose delivery and uptake via saturation transfer from hydroxyl protons [28] and has been translated, e.g., to head and neck cancer application at clinical 3 T systems [29]. DGE is often implemented using CEST or relaxation-based readouts (e.g., *T*_1rho_) and feasibility has been demonstrated in patients and healthy volunteers [30, 31]. DGE contrast is dominated by glucose delivery and intravascular (blood) contributions, and therefore, primarily reflects perfusion-/transport-related effects, rather than direct metabolic information. While these techniques are closely related to glucose-based DMI, they remain indirect assessments of glucose metabolism, which are more sensitive to motion and *B*_0_/*B*_1_ effects. In contrast, DMI provides direct spectral detection of multiple ^2^H-labeled tracers, i.e., glucose and downstream metabolites (Glx and lactate), enabling more specific separation of uptake and metabolism at the cost of lower sensitivity and the requirement of multinuclear capability of the MR system and dedicated RF hardware.

This review presents an overview of the latest advances in methodological development in signal acquisition, reconstruction, post-processing, and quantification for DMI over the last decade.

## Data acquisition

### Hardware

#### System modification for DMI capability

Non-proton-based MR techniques require MR systems that support multi-nuclear acquisitions as well as dedicated radiofrequency (RF) hardware including dual-tuned transmit/receive coils. However, multi-nuclear functionality, particularly for ^2^H is not supported by all MR vendors and scanners, yet, restricting widespread applications of DMI to few research sites.

To overcome these limitations, two research groups demonstrated hardware adaptations to enable ^2^H detection on MR systems without multi-nuclear option for ^2^H. By replacing the base frequency of a system-supported nucleus (e.g., ^17^O) with an external frequency generator and feeding the signal into the local oscillator transmit and receive boards, Seres Roig et al. and Ruhm et al. enabled ^2^H acquisitions on human 7 T [32] and 9.4 T [33] systems, respectively. No further hardware modification of the RF chain was required as Larmor frequencies between ^17^O and ^2^H nuclei are comparable (40 vs 45 MHz at 7 T, respectively) and sophisticated SAR simulations and measurements were performed by each group. In a similar fashion, Gursan et al. modified their 7 T MR system to enable DMI acquisitions by changing the gyromagnetic ratio from ^17^O to ^2^H in combination with a built-in four-channel receiver board from a 1 T system to convert ^2^H analog to digital signals [34]. However, to accelerate the adoption of DMI, stronger support from vendors will be essential.

#### Modified gradient filters

The implementation of DMI at clinical field strengths, such as 3 T, is challenging due to the inherently lower SNR and, additionally, can be affected by conflicting hardware constraints. Adamson et al. identified hardware-induced noise-like artifacts resulting from the gradient drivers, exhibiting harmonics in the frequency band of ^2^H resonances (18.9 MHz), which are inadequately filtered using vendor-provided gradient filters [35]. This introduced non-Gaussian noise that reduced spectral SNR by up to 25-fold. They addressed this issue by proposing a modified gradient filter with eightfold increased capacitors and by separating half of the cores for differential filtering. This significantly improved apparent SNR and represents an essential step forward toward more reliable DMI at clinical 3 T systems.

#### Radiofrequency coils (head)

As the availability of commercial ^2^H RF hardware is limited, many research groups rely on custom-built RF hardware for specific applications, i.e., dedicated head or body coils. Over recent years, many different coil designs were presented ranging from simple single loop ^2^H transmit/receive surface coils mainly used in preclinical studies [36, 37] to more sophisticated multichannel approaches. Volume coils, such as birdcage designs, provide uniform *B*_1_^+^ transmit field distribution with good homogeneity. Kaggie et al. developed a 4-rung ^2^H birdcage head coil capable of acquiring high-quality 3D DMI maps of the human brain at 3 T [38]. Similarly, Adamson et al. developed a quadrature transmit/receive birdcage coil tailored for brain DMI at 3 T [35], while Bednarik et al. employed a commercially available quadrature ^2^H birdcage head coil at 7 T [39]. Although volume coils offer excellent transmit field homogeneity across large volumes, their sensitivity is generally lower compared to surface coil arrays, particularly in the coil periphery. To address this, Seres Roig et al. and Ruhm et al. recently presented a similar design of a dual-tuned ^1^H/^2^H phased array RF head coil for 3D DMI at 7 T [32] and 9.4 T [33], respectively. Each design consisted of 8-channel proton and 10-channel ^2^H transceiver array operating in fixed circular polarization phase mode during transmission, to ensure relatively homogenous *B*_1_^+^ transmit field across the entire brain. A relatively low number of large receive elements provide high SNR for ^2^H signals in the brain periphery, while a 25–40% lower SNR drop was observed in the center of the brain.

#### Radiofrequency coils (body)

**Fig. 1:**
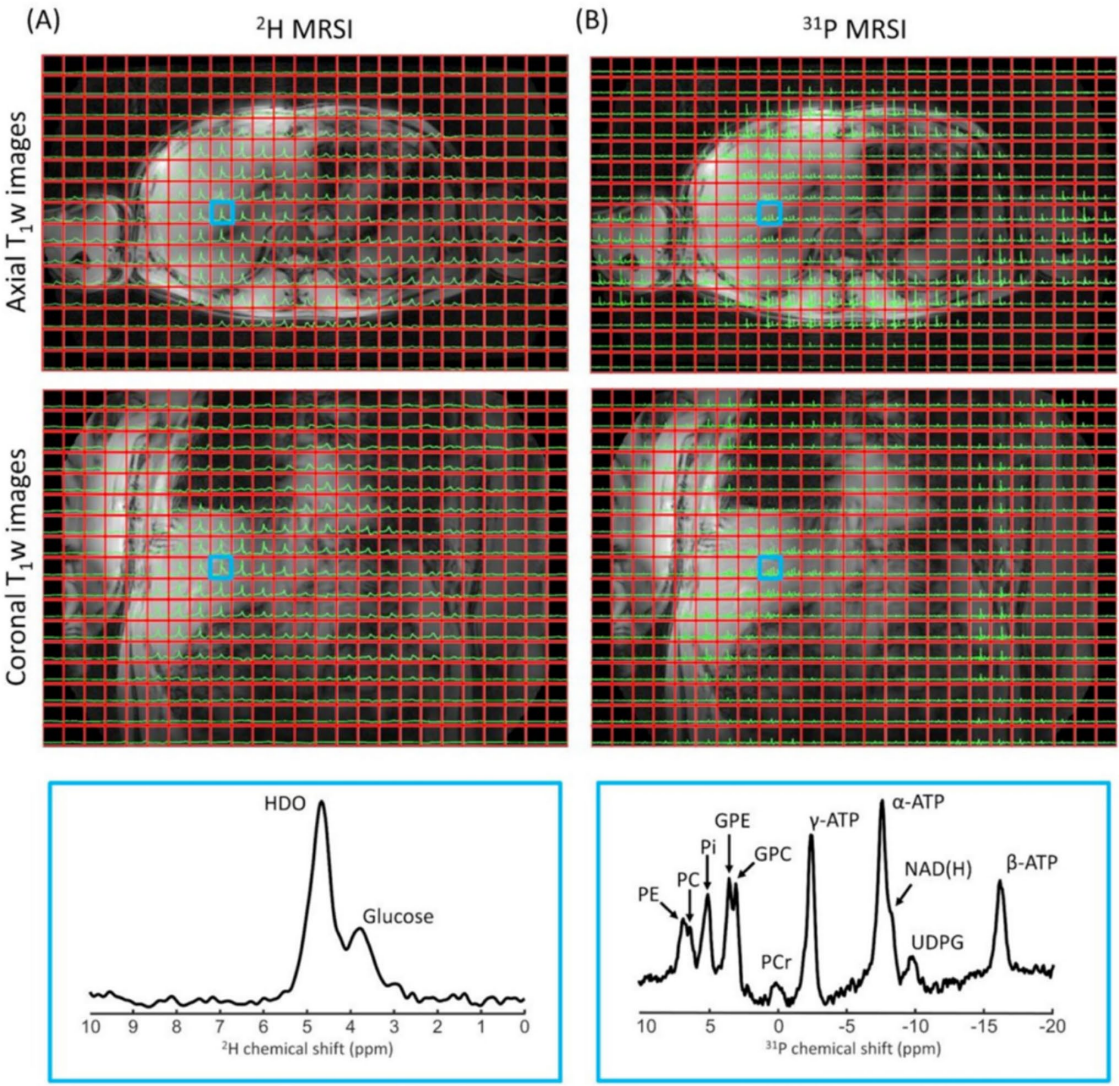
One slice of ^2^H 3D MRSI (**A**) and ^31^P 3D MRSI (**B**) data of the liver overlaid on the axial and coronal T1w images, together with enlarged spectra from a selected voxel (indicated in blue). ^2^H 3D MRSI data were acquired 90 min after oral administration of [6,6′‐^2^H_2_]‐glucose and the ^31^P 3D MRSI were acquired directly thereafter, without repositioning of the subject. ^31^P spectra were apodized with 20 Hz and spectra from muscle were clipped along the vertical axis in order to visualize the smaller signals in the liver. ^2^H/^31^P spectra were zero‐filled to 2048/512 points. UDPG, uridine diphosphate glucose (Figure reproduced with permissions from [40] licensed under CC BY 4.0)

As interest in DMI expands beyond neuroimaging, new hardware solutions have been developed to support abdominal and whole-body imaging. Gursan et al. developed a dual-tuned ^1^H/^2^H anterior/posterior body array coil consisting of four ^1^H fractionated dipole antennas (30.4 cm) combined with four ^2^H transmit/receive loops (12.65 × 27.65 cm^2^) enabling 3D abdominal DMI at 7 T [34]. Large loop diameters of ^2^H transmit/receive elements provide relatively homogeneous *B*_1_^+^ excitation across the entire abdominal volume. To extend anatomical coverage, the same group recently introduced a novel dual-tuned ^2^H/^31^P whole-body transmit coil with a 60 cm diameter and 40 cm length [40]. This shielded 24-rung high pass birdcage design was embedded in the scanner bore of the MRI system. The feasibility was demonstrated with separate ^2^H and ^31^P MRSI acquisitions of the human brain, abdomen and lower leg muscles, see Fig. 1. For signal reception, a commercial dual-tuned eight channel ^2^H/^31^P receive array was used with additional eight fractioned transmit/receive ^1^H dipole antennas for anatomical imaging and *B*_0_ shimming.

Other groups have employed flexible or form-fitted commercial coil solutions. Wodtke et al. and Mclean et al. used a flexible transmit/receive ^2^H coil (RAPID Biomedical, Germany) to perform abdominal DMI at 3 T following oral tracer administration, successfully mapping temporally resolved ^2^H-glucose and ^2^H-water signals [41, 42]. Niess et al. utilized a form-fitted dual-tuned two-channel ^2^H, one-channel ^1^H transmit/receive body array for high-resolution 3D renal DMI at 7 T [43].

### Acquisition schemes (sequences)

Due to short transversal relaxation times, the vast majority of DMI studies employ pulse-acquire sequences based on free induction decay (FID), typically followed by spatial localization using either the effective field-of-view of a local surface coil or 2D/3D phase encoding with Cartesian k-space sampling. Slice-selective excitation or refocusing can be constrained by the available gradient amplitude of the MR system and the chosen RF pulse bandwidth/duration because the low gyromagnetic ratio of deuterium requires 6.5-fold higher-gradient amplitudes to achieve similar slice/slab thickness compared to ^1^H MRS. Therefore, most human studies rely on non-selective excitation combined with a 3D readout sampling scheme.

The short *T*_1_ relaxation times of ^2^H nuclei enable shortening repetition times below 300 ms, which allows for 3D mapping with clinically acceptable spatial and temporal resolution. At clinical field strengths, reported nominal voxel volumes in DMI studies of the human brain range from approximately 33 ml [38] to 14 ml [35, 44, 45]. Improved resolutions have been achieved at higher field strengths, i.e., 8 ml (or as small as 1 ml with reduced FOV) at 4 T [16, 27] and 2 ml (1 ml with reduced FOV) at 7 T [27, 32, 39, 46]. One study at 9.4 T achieved 3 ml [33] in 10 min acquisition duration enabling dynamic DMI with high spatial and temporal resolution. Total acquisition times for these datasets vary between 7 and 30 min, depending on matrix size and number of averages needed to achieve sufficient SNR, especially at clinical field strengths.

To reliably capture the metabolic dynamics of deuterium labeled substrates over time immediately following tracer administration, temporal resolutions of 10 min or less per time point are generally required. This is particularly important for quantitative estimation of metabolic turnover rates [47, 48]. However, conventional Cartesian k-space sampling is inherently slow, which is a major constraint to achieve high-resolution dynamic DMI experiments without sacrificing on temporal resolution.

**Fig. 2:**
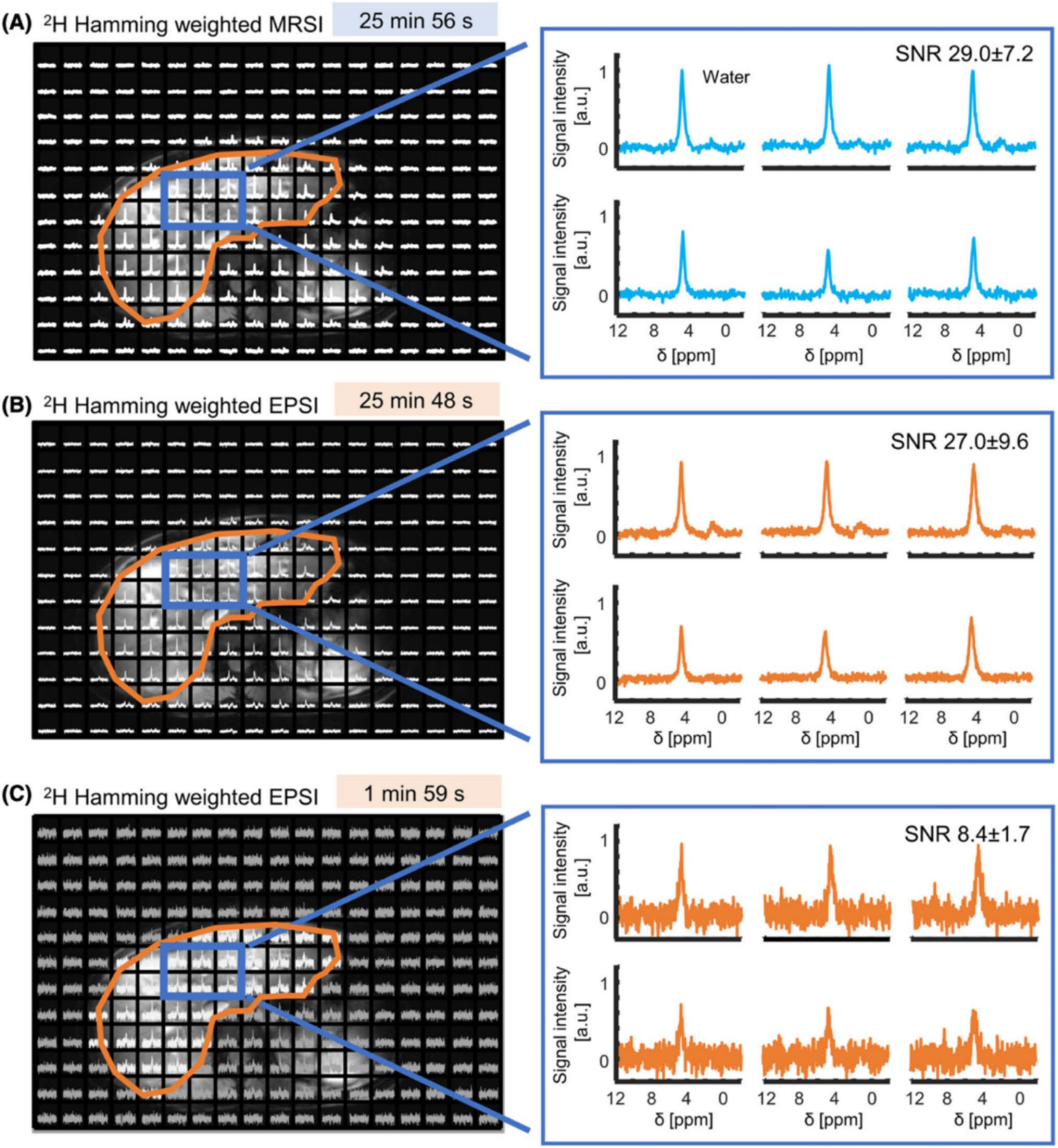
Three‐dimensional data sets of the human liver at natural abundance, acquired with both Hamming‐weighted MRSI (**A**) and EPSI acquisitions (**B**, **C**). The left panels show overlays of the ^1^H‐MRI images (Dixon) and the ^2^H spectra. The liver is contoured in orange in the left panels. The right panels highlight the selected six voxels located in the liver (left panels, blue box), and SNR was calculated for 18 voxels from the three middle slices. Measurement parameters: nominal voxel size *V*_nom_ = 8 ml, zero‐filling to 512 points, baseline correction was applied, but no line broadening and no de-noising (Figure reproduced with permission from[55] licensed under CC BY 4.0)

Time-efficient k-space sampling can be achieved with spatial–spectral encoding strategies to simultaneously assess spatial and spectral information such as Echo-planar Spectroscopic Imaging (EPSI) [49], spirals [50], rosettes [51, 52], or concentric rings [53, 54]. While no intrinsic SNR advantage is obtained for equal scan durations, such sampling schemes can substantially accelerate acquisitions and therefore, provide a flexible tradeoff between SNR efficiency and measurement time. This has been recently implemented for abdominal DMI studies, e.g., Nam et al. traded a 3.6-fold SNR penalty for a 13-fold reduction in acquisition time for liver DMI using EPSI, enabling full metabolic imaging in 2 min [55], see Fig. 2.

**Fig. 3:**
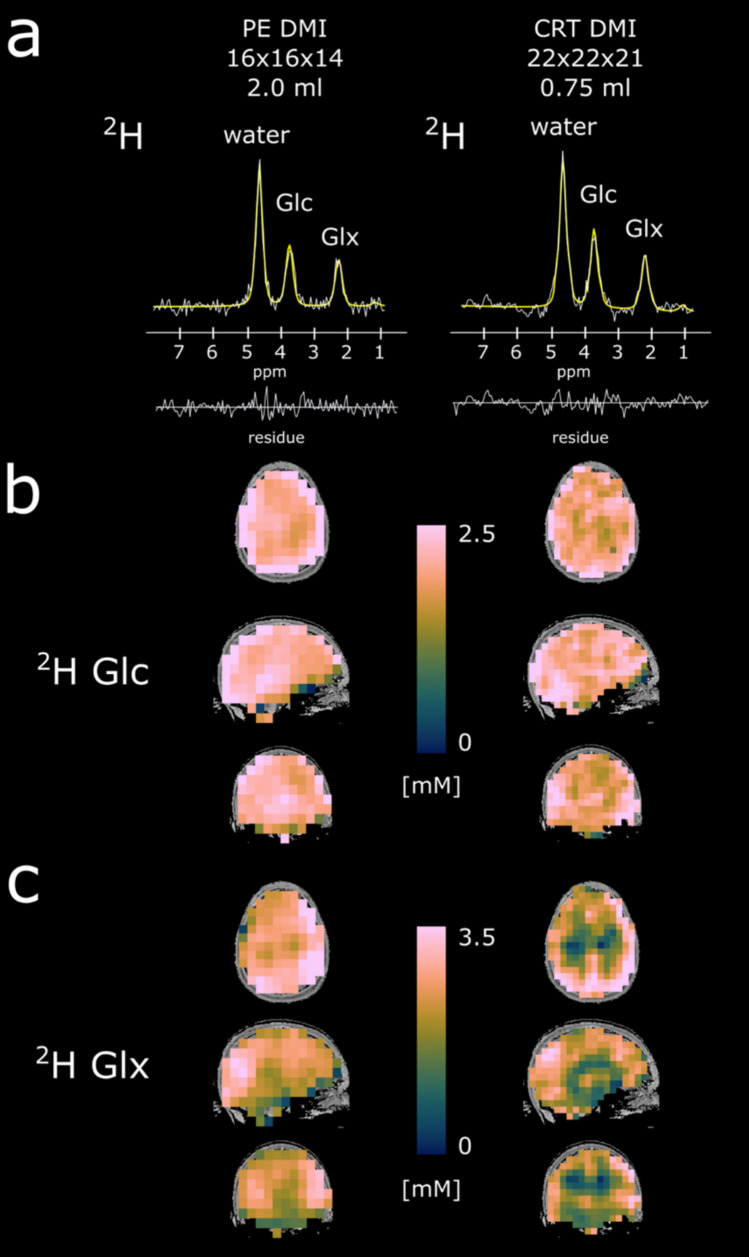
Comparison of spatial resolution between 2 ml and 0.75 ml for phase-encoded deuterium metabolic imaging (PE DMI, left column) and concentric ring trajectory DMI (CRT DMI, right column), respectively. Representative sample spectra, spectral fits and residues (**a**) of the last DMI scan (63 and 70 min after oral administration of deuterium labeled glucose) featuring resonances of ^2^H water, ^2^H glucose (Glc) and ^2^H glutamate + glutamine (Glx). The 3D metabolic maps of Glc (**b**) and Glx (**c**) from one representative volunteer. Faster metabolism in GM compared with WM, that is, increased Glx concentrations are more pronounced for CRT maps (Figure reproduced with permissions from[56] licensed under CC BY 4.0)

Similarly, Niess et al. achieved a 2.6-fold increase in spatial resolution in the human brain attaining isotropic nominal voxel volumes of 0.75 ml using a combination of concentric ring trajectory (CRT) sampling and advanced de-noising during post-processing to maintain sufficient SNR [56], see Fig. 3. This sampling scheme was employed for abdominal measurements by the same group for dynamic high-resolution 3D renal DMI in humans at 7 T with spatial resolutions of 1.8 ml and 0.9 ml to assess the metabolic dynamics of orally-administered ^2^H-glucose and ^2^H-water, respectively [43]. These advances illustrate the growing potential of accelerated k-space sampling schemes in making DMI faster and more clinically practical.

#### Improving SNR of DMI

The successful implementation of DMI at widely available clinical field strengths and its adoption into clinical practice largely depends on further improvements in SNR to increase spatial resolution and reliably detect lower metabolite concentrations, e.g., relatively shortly after oral glucose administration. Such advancements are crucial for DMI to compete with more established metabolic imaging modalities such as PET.

A promising approach to address this challenge is the use of the balanced steady-state free precession (bSSFP) principle, which is a well-established method in clinical MRI to maximize SNR efficiency [57]. Unlike conventional spoiled MR sequences, bSSFP achieves a steady-state of transversal magnetization by employing short repetition times. The SNR gain is best for $T_{{\mathrm{R}}} \ll T_{2}$ and when the ratio *T*_2_/*T*_1_ is high. The absence of spoiler gradients between subsequent RF excitations preserves phase coherences and maximizes SNR per unit time. Variants of this method are commonly known as FISP, TrueFISP or CISS [58]. Applying this principle to MRSI is not straightforward as short repetition times restrict the acquisition window, which is important to allow sufficient phase evolution to happen and is required to separate different chemical resonances reliably. Nevertheless, several groups have recently demonstrated that bSSFP can be implemented for DMI. In preclinical studies at 15.2 T, Peters et al. and Montrazi et al. demonstrated that multi-echo bSSFP and CSI-based bSSFP enabled approximately a fourfold increase in SNR for ^2^H-labeled metabolites compared to traditional spoiled FID-based MRSI acquisitions [59–61]. In these rodent studies, increased SNR allowed for a detection of ^2^H-glucose, ^2^H-lactate, and ^2^H-water with improved spatial resolution. At 7 T, Frese et al. applied a similar bSSFP DMI approach in the human brain using a modified MRSI sequence based on 3D CRT readout [62]. They reported a threefold increase in SNR relative to spoiled FID-MRSI using a similar readout, which allowed for a twofold increase in spatial resolution for whole-brain maps of ^2^H-labeled compounds. To overcome the challenge of limited spectral resolution, all groups implemented an extended version of the iterative decomposition of water and fat with echo asymmetry and least-squares estimation (IDEAL).

**Fig. 4:**
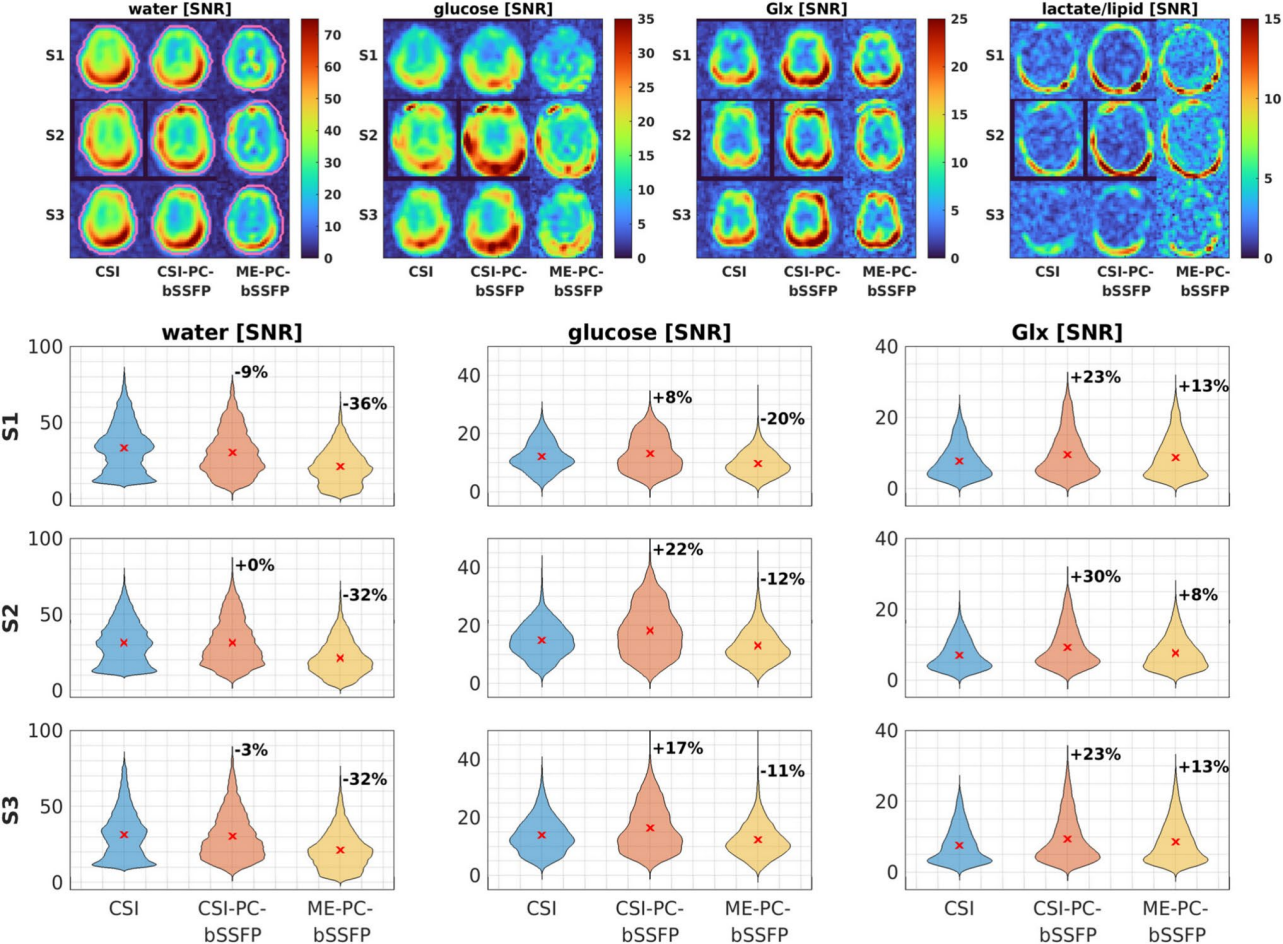
In vivo SNR analysis for all four deuterated metabolites in three subjects. An exemplary axial slice of the metabolite maps (top) and the SNR distribution over the entire brain (bottom) are shown for the three different acquisition methods. The whole‐brain ROIs are depicted as overlaid magenta contour lines in the water SNR image. The average SNR is illustrated as a red cross and the percentage change of the average SNR relative to the standard chemical shift imaging (CSI) acquisition is depicted next to the respective violin plots. The interleaved acquisitions are averaged to minimize the influence of metabolic dynamics. The SNR maps from the CSI phase‐cycled (PC)‐bSSFP and multi‐echo phase‐cycled bSSFP (ME‐PC‐bSSFP) acquisitions are normalized with their corresponding point spread function (PSF) volume relative to the standard CSI, resulting in normalization factors of 0.94 and 1.52, respectively (Figure reproduced with permission from [63] licensed under CC BY 4.0)

Valsala et al. applied different variants of bSSFP sequences for whole-brain DMI at 9.4 T and compared the SNR performance and improved robustness against *B*_0_ in-homogeneities for acquisition schemes with introduced phase-cycling [63], see Fig. 4.

These studies demonstrate that bSSFP-based DMI, particularly when combined with advanced sampling strategies, such as spatial-spectral k-space encoding, holds substantial promise for improving image quality and metabolic specificity in both preclinical and clinical settings. Nonetheless, careful attention to *B*_0_ in-homogeneities remains critical as bSSFP sequences are particularly vulnerable to off-resonance effects.

#### Temporal multinuclear interleaving

Advances in k-space encoding strategies have significantly reduced the acquisition times required for individual DMI datasets, enabling higher spatial resolution within clinical feasible sampling durations. However, despite these improvements, capturing the metabolic dynamics following the administration of ^2^H-labeled substrates typically requires extended measurement windows of 60–120 min. Prolonging the duration of an MR session by up to 2 h is impracticable for clinical implementation. Therefore, long dynamic DMI protocols targeting full kinetic modeling will likely remain primarily a research tool, particularly when metabolic imaging needs to be accompanied with standard MRI protocols for anatomical and structural assessments. This limitation becomes especially relevant in clinical settings where MRI examinations are already lengthy, e.g., in neuro-oncology, where comprehensive multi-parametric protocols are routinely acquired. In such scenarios, approaches that can extract dynamic metabolic information simultaneously without further extending scan time could be particularly attractive.

**Fig. 5:**
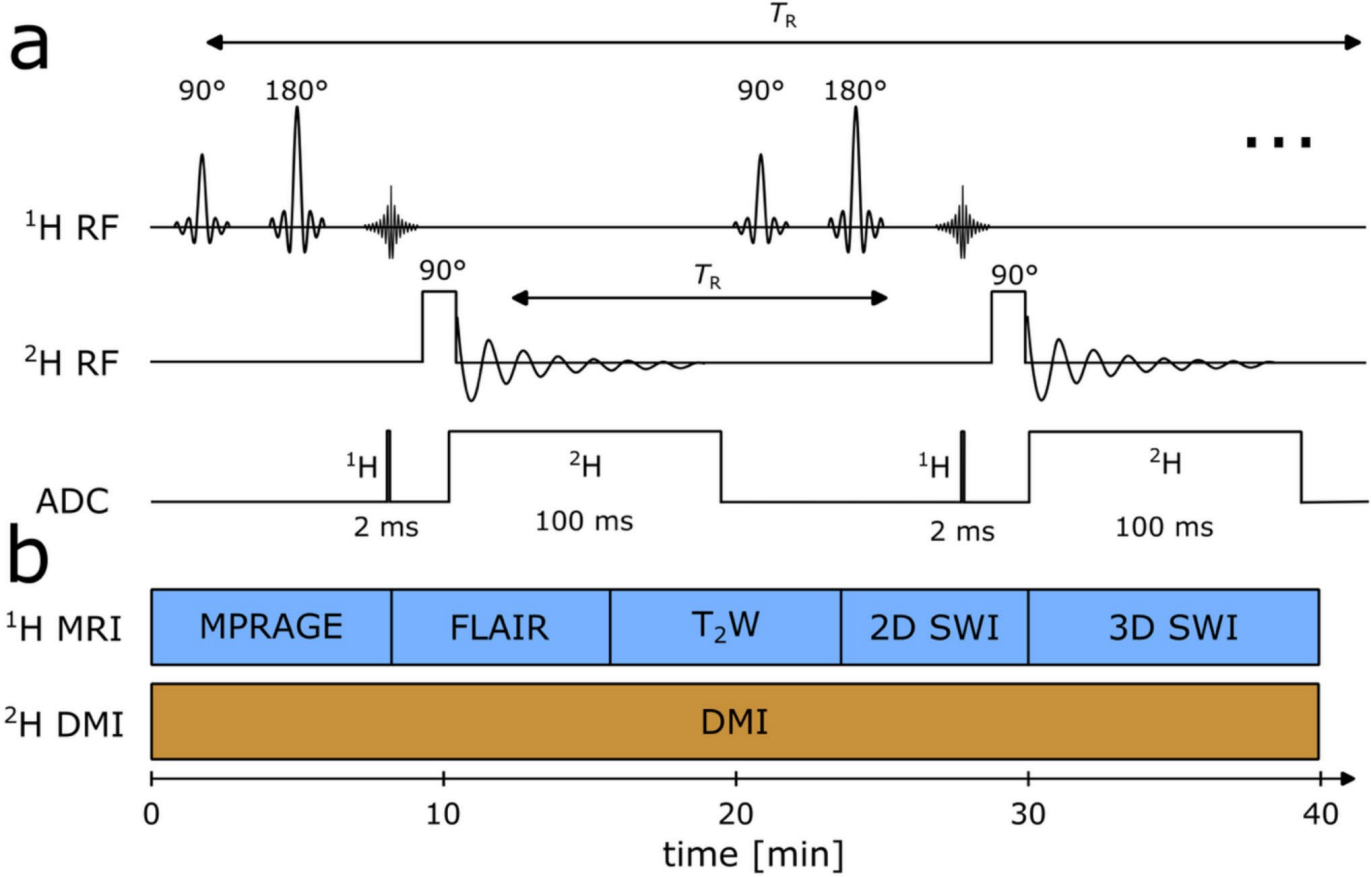
Simplified illustration of temporal interleaving ^2^H-FID-MRSI sampling (e.g., 100 ms readout) within time delays of exemplary ^1^H MRI acquisitions (e.g., 2 ms readout). **a** Liu et al. applied a similar principle to an entire clinical ^1^H MRI protocol including MPRAGE, FLAIR, T2W, 2D SWI and 3D SWI. Multi contrast whole-brain ^1^H images together with metabolic information provided by simultaneously acquired whole-brain DMI [65] within 40 min (**b**)

A promising solution was recently proposed by Liu et al. demonstrating a temporal multinuclear interleaving strategy that enables simultaneous acquisition of conventional proton (^1^H) MRI sequences alongside dynamic whole-brain DMI data, without increasing overall scanning time [64]. Substantial differences in Larmor frequencies between ^1^H and ^2^H (approximately 300 MHz vs. 45 MHz at 7 T, respectively) ensure no perturbation of magnetization or signal evolution between both nuclei due to consecutive multinuclear RF excitation. As a result, independent excitation and acquisition blocks can be interleaved within the same pulse sequence framework without major interference enabling almost simultaneous data sampling. This concept was demonstrated using a comprehensive clinical MRI protocol that included *T*_1_-weighted MPRAGE, *T*_2_-weighted turbo spin echo, FLAIR and both 2D and 3D susceptibility-weighted imaging (SWI) sequences [65]. Whole-brain 3D DMI data were acquired in parallel during the relaxation intervals of the ^1^H sequences, allowing simultaneous assessments of both structural and metabolic information, see schematic illustration in Fig. 5. No degradation in image quality was observed for either modality using a parallel acquisition compared to separate consecutive acquisitions, thereby validating the technical feasibility and clinical utility of this approach. Temporal multinuclear interleaving represents a significant step toward the integration of DMI into clinical routine workflows by eliminating the need for increased scanning duration and enhancing patient comfort.

#### ^1^H-observed (indirect) detection (quantitative exchange label turnover)

**Fig. 6:**
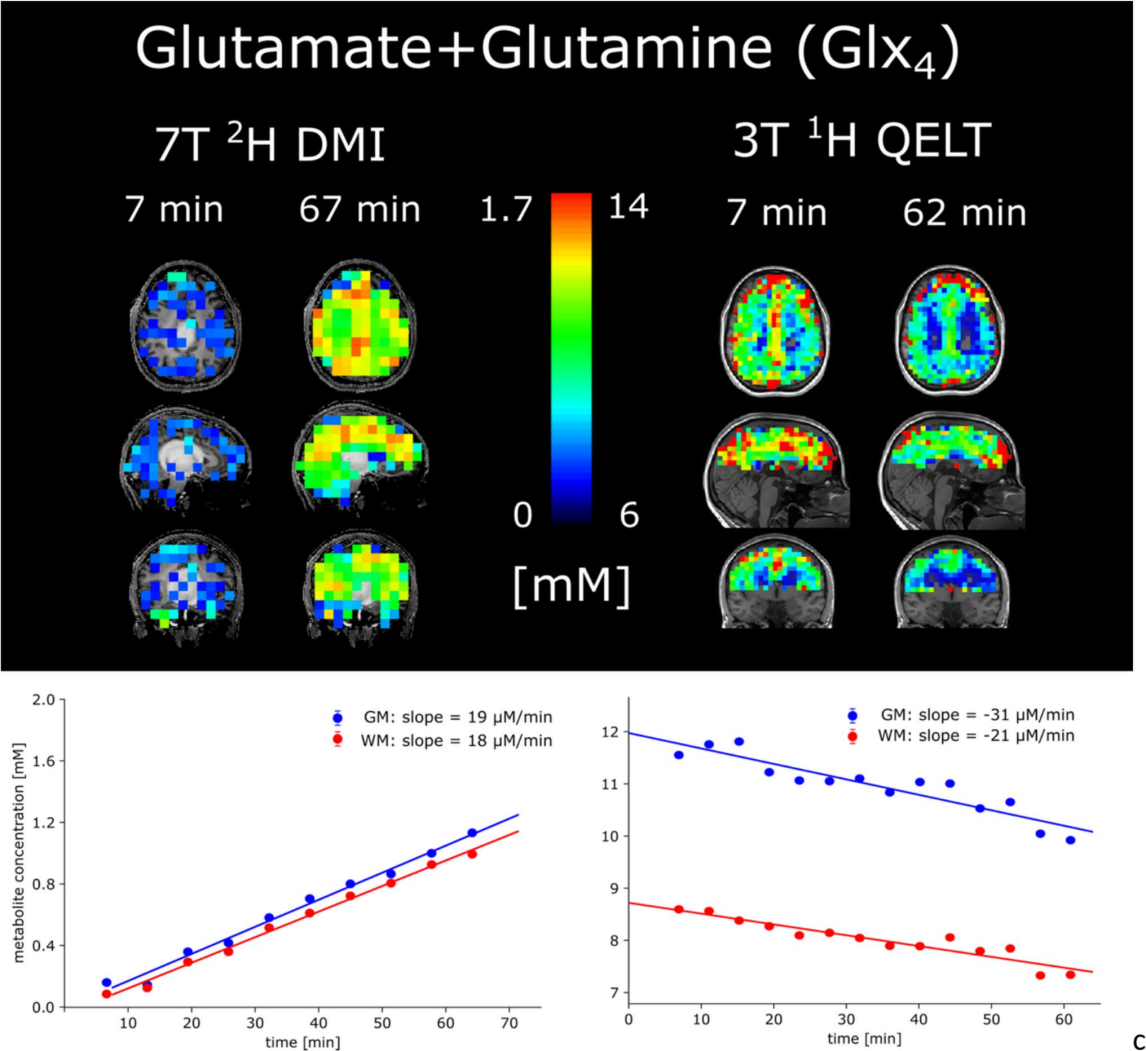
Representative 3D (glutamate + glutamine) Glx_4_ maps (top) and corresponding averaged time courses over GM and WM dominated regions (bottom) of direct ^2^H-DMI(left) and indirect ^1^H-QELT (right) resonances acquired 7 min and 67/62 min after deuterium labeled glucose administration. The signal intensity increase/decrease of respective ^2^H and ^1^H resonances is clearly visible. Missing voxels in the Glx_4_ metabolic maps do not contain a value (NaN: not a number) (Figure reproduced with permission from [46] licensed under CC BY 4.0)

DMI is a non-proton MR method requiring MR systems with multinuclear option and dedicated RF equipment such as dual-tuned coils to allow localizer and structural imaging and shimming. Additionally, inherently low SNR and low natural abundance limit the achievable spatial resolution of DMI at clinical field strength. Quantitative exchange label turnover has recently been proposed as an alternative to detect accumulation of ^2^H-labeled substrates in vivo indirectly via conventional ^1^H MRS or MRSI methodology. For conventional brain studies at rest, ^1^H MRS/MRSI detect steady-state concentrations of high-energy metabolites, e.g., glutamate, an excitatory neurotransmitter, which is oxidatively metabolized in the TCA cycle from glucose and later converted to glutamine. Administration of ^2^H-labeled substrates, such as [6′,6]-^2^H glucose, leads to accumulation of ^2^H-labeled molecules in the total (steady) pool of glutamate and glutamine. The replacement of ^1^H-labeled by ^2^H-labeled compounds leads to a reduction of ^1^H-detectable signal, which can be detected using ^1^H-MRS/MRSI methods. This has been shown by Rich et al. in a preclinical setup using ^1^H-MRS [66] and later by Bednarik et al. and Cember et al. in the human brain at 7 T using whole-brain FID-MRSI [39] and PRESS-MRSI [67], respectively. Correlations between direct (DMI) and indirect (QELT) deuterium detection were observed by the same group acquiring 3D ^2^H-MRSI at 7 T and 3D ^1^H-MRSI at clinical 3 T, see Fig. 6. After oral administration of ^2^H-labeled glucose, increasing concentration estimates of directly detected ^2^H resonances of glucose and combined glutamate + glutamine (glx) using DMI were not different from decreasing concentration estimates of conventional detected ^1^H Glc and Glx using QELT [46]. A similar comparison was performed by Ruhm et al. using single-voxel ^1^H-MRS compared with 3D ^2^H-MRSI of the human brain at 9.4 T [68]. However, care must be taken to achieve robust data quality of ^1^H MRS/MRSI acquisitions to reliably assess concentration changes between 10 and 30%. In this respect, QELT is inherently more vulnerable to confounds than direct DMI as it quantifies relatively small signal decreases on top of larger ^1^H metabolite resonances and therefore is inherently more sensitive to magnetic field drifts, lipid contamination, changes in lineshape and motion-induced baseline instabilities. Hence, efficient suppression or removal of strong lipid signals is required, which can overlap and bias the accurate quantification for the metabolites of interest. Especially the presence of uncorrected motion effects can lead to contamination from strong lipid signal, e.g., from subcutaneous fat outside of the brain. To mitigate those challenges and minimize spectral artifacts, prospective motion correction using interleaved volumetric navigators [69, 70] and lipid removal strategies [71, 72] have been proposed. Overall, QELT is highly attractive because it can be implemented on standard clinical ^1^H hardware, but its quantitative reliability depends on exceptionally stable ^1^H MRSI across time points. In addition, QELT typically assumes temporally stable metabolic pool sizes since label incorporation is derived from relative signal decreases. In contrast, DMI directly observes labeled ^2^H resonances in a comparatively sparse spectrum, while it remains more strongly constrained by SNR and the requirement of multinuclear capabilities of the MR system plus dedicated RF hardware. Consequently, the two approaches should be viewed as complementary, with QELT prioritizing accessibility and DMI prioritizing specificity of the metabolic readout. These results highlight the significant potential of ^1^H-observed deuterium detection on clinical MR systems, especially in environments with limited access to ultra-high field scanners and dedicated RF hardware.

## Data reconstruction

### Model-based data reconstruction

Traditional DMI acquires phase-encoded k-space data and reconstructs images by a simple Fourier transformation. While straightforward, this approach fails to exploit redundancies across spectral, spatial, and temporal domains. Additionally, DMI has several advantages in that regard over ^1^H-MRSI. The signals from water and lipids are not much higher than the signals of interest, in contrast to ^1^H-MRSI, where they are several orders of magnitude higher. Therefore, low-rank approximations will not be dominated by these signals, and they do not need to be removed in post-processing prior to advanced reconstructions. Second, the ^2^H-spectra are sparser with fewer resonances, which are not overlapping. This makes DMI data have a lower rank, and the sparseness can be exploited in Compressed Sensing-like reconstructions. Third, DMI usually has more dimensions since it is often measured dynamically. By that, data redundancy can be exploited more easily. Finally, *B*_*0*_ in-homogeneities affect DMI data less due to the lower gyromagnetic ratio of ^2^H. Thus, spectral alignment prior to low-rank reconstructions is less important. However, since DMI usually has short TR’s and low spatial resolutions, additional accelerations (apart from spatial-spectral encoding) are usually not necessary. Therefore, e.g., methods like SPICE or Compressed Sensing are not applied to accelerate the measurements, but to de-noise data.

Due to all these advantages, several studies have therefore focused on model-based reconstructions, where prior information constrains the inverse problem to yield higher fidelity from noisy data.

Li et al. introduced a subspace-based AI reconstruction that combines low-rank approximations based on spin physics simulations from the SPICE framework with AI learned spectral–temporal manifolds [73]. Their model represents the signal as partially separable functions, where one depends on space and spectral frequency, and the other on space and kinetic time. Both of these functions are modeled to be partially separable. Spectral basis functions were generated from estimated lineshape variabilities, while temporal basis functions were pre-trained using high-SNR training data. Additionally, deep auto-encoders were trained to capture low-dimensional manifolds of the spectral and temporal variations using physics-based simulated training data. Both the subspace model and the deep auto-encoders were combined in an optimization problem. Spatial constraints were incorporated after subspace fitting, producing robust metabolic maps even in in vivo rat data. The reconstruction jointly enforced data consistency, low-rankness, and proximity to learned manifolds, yielding minimal bias and four- to five-fold RMSE reduction relative to conventional methods.

This framework was recently extended to DMI in humans using a multi-channel dual-tuned ^2^H/^1^H coil with 4 channels for each nucleus [74]. The reconstruction was extended with motion and *B*_0_ correction, WSVD coil combination, and metabolite-specific subspace fitting. Additionally, *t* quantitative metabolic fluxes were calculated using metabolic modeling. The result was a two- to three-fold SNR gain versus standard reconstructions and precise metabolic flux quantification (CMR_Glc_, *V*_TCA_) with strong gray-matter dependence.

Nam et al. also used the SPICE framework to reconstruct human liver ^2^H-MRSI [75]. Simulations using segmented digital phantoms confirmed that SPICE substantially reduces normalized RMSE and Cramér–Rao bounds compared with Fourier reconstruction, even under retrospective acceleration (under-sampling factor 1.3). In two human subjects, a SPICE-like reconstruction achieved higher SNR and lower uncertainty for glucose and downstream metabolites. These results validate SPICE as a robust framework for low-SNR metabolic imaging in DMI, capable of leveraging temporal correlations and k-space structure for accurate dynamic reconstructions.

Christensen et al. also used low-rank approximations for their reconstructions of ^23^Na, ^2^H, and ^13^C MRSI data [76], while comparing two tensor-low-rank methods: GL-HOSVD and tMPPCA de-noising. GL-HOSVD performs a global higher-order SVD followed by localized refinement, while tMPPCA recursively thresholds singular values based on the noise distribution. Both methods improved reproducibility and quantitative accuracy, but tMPPCA required fewer parameters and was substantially faster. These studies demonstrate that data-driven tensor de-noising provides a principled, non-heuristic means to separate signal from noise across spatial, spectral, and temporal domains.

**Fig. 7:**
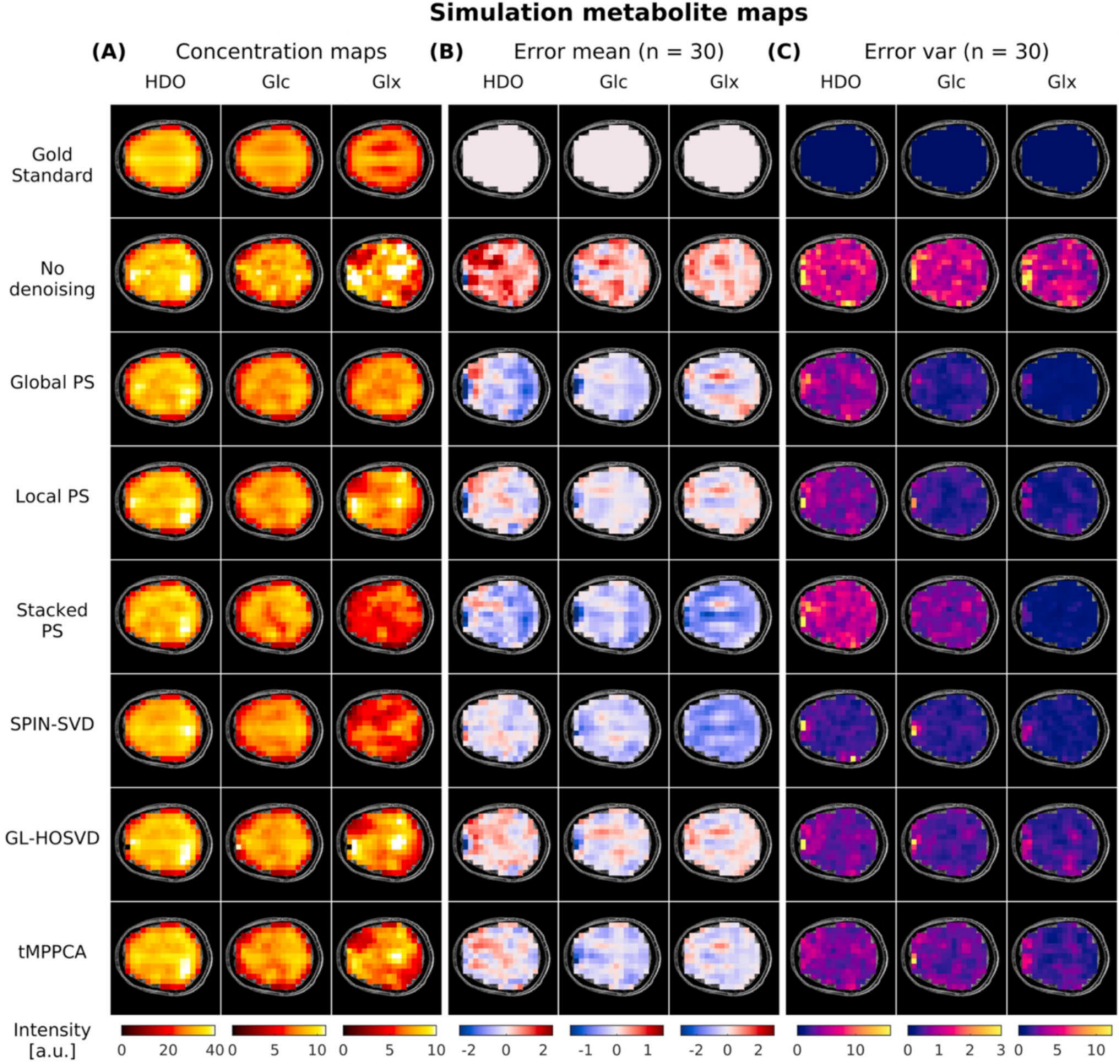
**A** Fitted maps of HDO, Glc, and Glx after applying different de-noising methods to the 2H‐MRSI simulation for a single noise instance. Voxel‐wise **B** mean and **C** variance of the error compared with the noiseless gold standard maps, calculated over 30 noise realizations. All maps show the final repetition of the simulated 2H‐MRSI data. The input Gaussian noise amplitude (before de-noising) corresponds to spectral SNRHDO = 9, SNRglc = 4 and SNRglx = 1.7. SPIN‐SVD performed best for HDO, resulting in low bias and variation, but introduced bias for Glx as shown by the overall negative mean error map. SPIN‐SVD and Local PS resulted in low bias and variance for Glc. All methods introduced distinct residual patterns in the Glx mean error maps, indicating GM‐to‐WM Glx contrast loss. GL‐HOSVD and tMPPCA resulted in low bias but higher error variance for Glx (Figure reproduced with permission from [77] licensed under CC BY 4.0)

Duguid et al. also compared matrix- and tensor-based low-rank de-noising approaches—global, local, and stacked partial separability (PS), SPIN-SVD, GL-HOSVD, and tMPPCA—on simulated and in vivo ^2^H-MRSI data [77]. SPIN-SVD (Spectral and Temporal INtegration for SVD), which mixes the temporal and spectral dimensions before decomposition, preserved spatial heterogeneity and lesion contrast best in simulations see Fig. 7. In vivo, SPIN-SVD, GL-HOSVD, and tMPPCA performed comparably, maintaining gray- and white-matter contrast while efficiently suppressing noise.

**Fig. 8:**
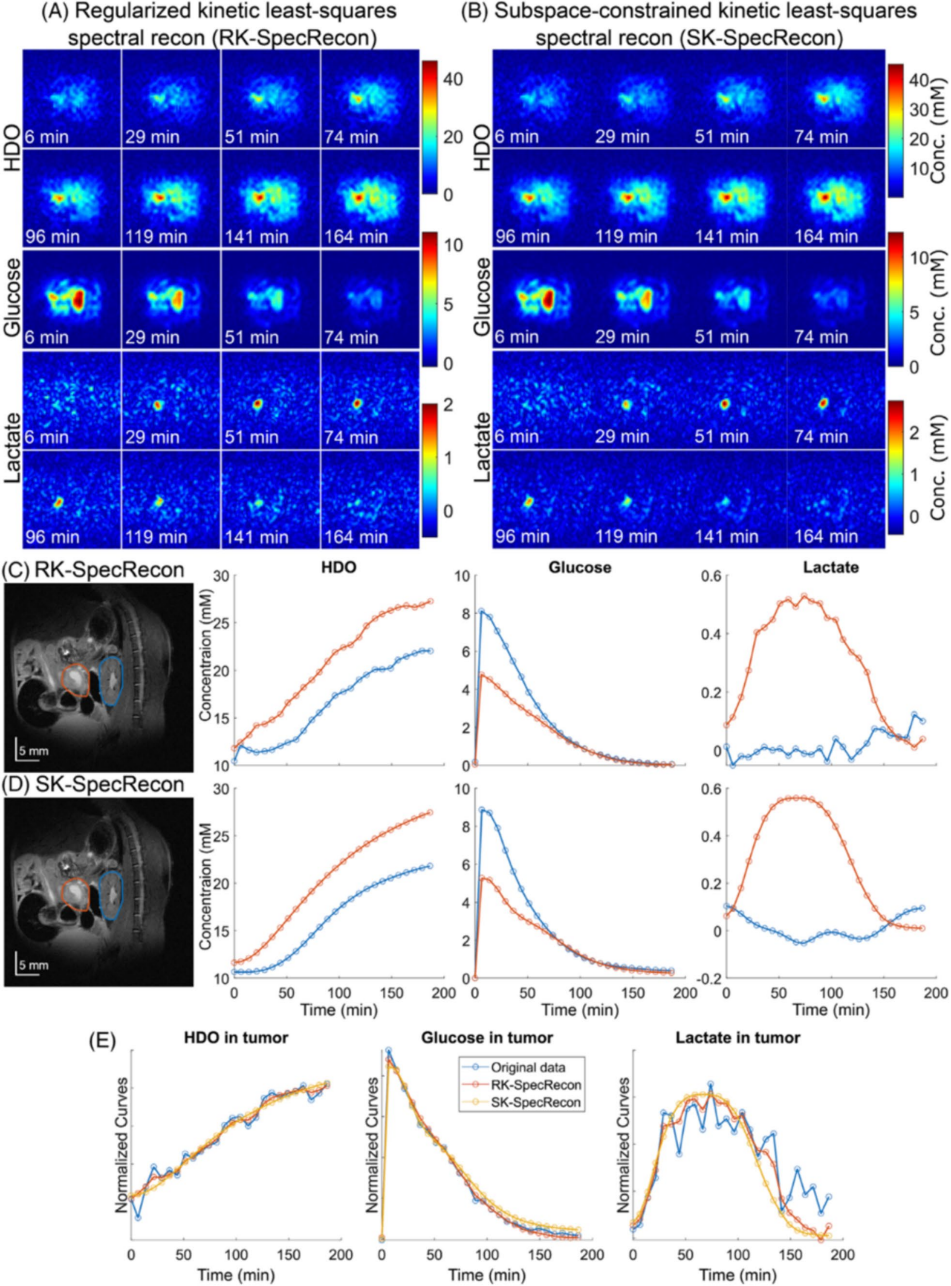
Comparison between the basic IDEAL and a subspace‐constrained + CoSeM de-noising reconstruction, performed on the same data. Lactate is produced to 0.3 mM levels or less in the tumor region; it is observable if using the latter reconstruction, but would be lost with the original ME‐bSSFP processing of Ref. [17]. Other features arising in the de-noised lactate maps, as well as the slightly negative values arising in the ensuing kinetic plots, indicate the errors of detection of the experiment (Figure was reproduced with permission from [59] licensed under CC BY NC ND 4.0)

A unique combination of low-rank and IDEAL reconstruction was used by Montrazi et al. [59]. They used a multi-echo bSSFP sequences with the IDEAL approach to jointly reconstruct metabolite concentrations and *B*_0_ maps from k-space data and modeled temporal kinetics as a sum of SVD-derived basis functions (“SK-SpecRecon”). By that, they achieved temporally smooth and physically plausible metabolite kinetics. Spatial regularization by compressed sensing (“CoSeM”) or block-matching filtering (BM3D) enhanced SNR without blurring, outperforming Gaussian filtering. This combination of multi-echo encoding and subspace reconstruction demonstrates the power of model-constrained approaches to extract quantitative information directly from encoded k-space, see Fig. 8.

### 2D-fitting

Another approach to exploit redundancy across spectral and temporal dimensions is 2D fitting. Instead of sequential fitting first along the spectral and then along the kinetic time dimension, 2D fitting models all spectra across all time points simultaneously, capturing shared information between them.

This concept was demonstrated by Tal [78] and Clarke et al. [79] within the FSL-MRS framework. Although Clarke et al. did not apply it to DMI, they showed in simulations and in vivo mouse and human data (functional, GABA-edited, and diffusion MRS) that 2D fitting reduces RMSE compared to independent spectral and temporal fitting.

**Fig. 9:**
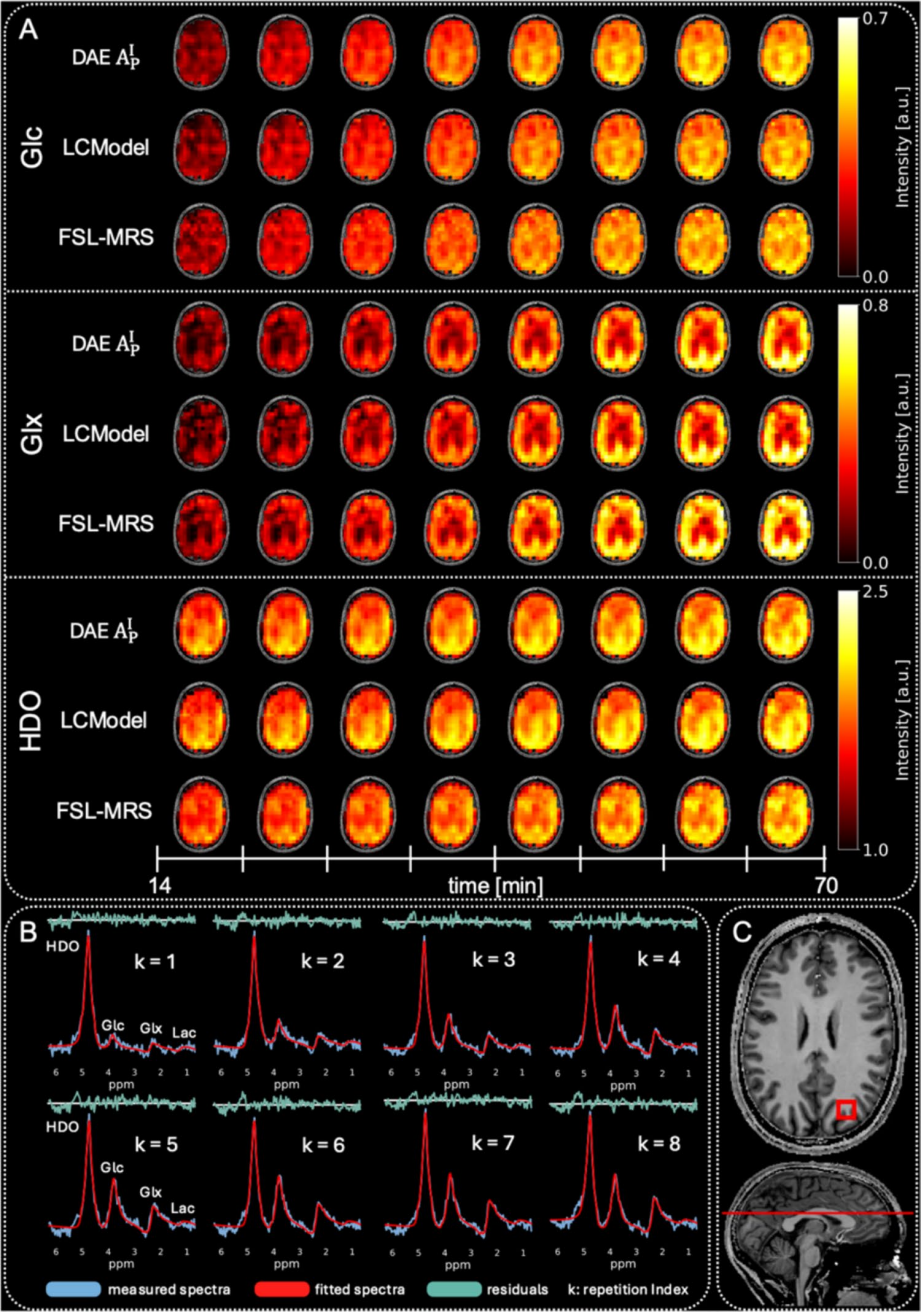
**A** Metabolite maps of the three major metabolite signals (Glc, Glx, HDO) across all eight time points for one subject from the in vivo test dataset, fitted by DAE instance *A*^*I*^_*P*_ and the reference methods LCModel and FSL-MRS. **B** Example spectral time course with corresponding DAE fits and residuals. The index *k* indicates the position of each spectrum in the time series, and metabolite labels identify the spectral peaks. **C** *T*_1_-weighted anatomical image with a red box indicating the voxel location of the time course and a red line marking the position of the presented metabolite maps (Figure was reproduced with permission from[80] licensed under CC BY 4.0)

The first application of 2D fitting to DMI was recently reported by Osburg et al. [80], where a deep auto-encoder was implemented that jointly models spectral and temporal variations in latent space, representing parameters, such as metabolite concentrations, frequency shifts, line-shape distortions, baselines, and temporal evolution functions (logistic, linear, or constant). In synthetic data, their method achieved the lowest RMSE compared with LCModel and FSL-MRS 2D fitting, and showed strong agreement with both for in vivo human data. Notably, it was also far more efficient—processing a dataset in 0.5 s, versus 17 min for LCModel and 20 min for FSL-MRS, see Fig. 9.

## Analysis, modeling, and quantification

As an MR-visible isotope, ^2^H has also the advantage of being present in very small natural abundance [81] (0.0156%), similar to the situation encountered for ^13^C, which enables the follow-up of the labeled isotope along the metabolic pathways of the administered substrate, with minimal background signal contamination. Thermally polarized dynamic ^13^C MRS applied in preclinical and clinical research has enabled the study of brain energy metabolism and its coupling to neurotransmission, providing important insights into mechanisms supporting brain function [82–85]. Besides the rich information provided by ^1^H MRS(I) on the neurochemical profile, representing metabolic steady-state balance of the underlying biochemical network, X-nuclei labeling experiments can offer kinetic information on the reaction rates, ideally in terms of metabolic fluxes (in micromole/g/min). This enables quantitative estimates of the activity of specific biochemical pathways, such as the TCA cycle, neurotransmitter cycling or glycolysis. It seems therefore logical to exploit this aspect of ^2^H MR, coupling concomitant infusion of ^2^H-labeled tracers with dynamic ^2^H MRSI to probe metabolism. ^2^H MRSI can be performed as a steady-state acquisition after administration of a deuterated substrate, providing information on active metabolic pathways in the tissue of interest, or as for dynamic ^13^C MRS, as a dynamic measurement following a bolus administration or continuous infusion of ^2^H-labeled substrates, providing information on the magnitude of the metabolic fluxes involved in those pathways.

### Quantification

The first step in metabolic map quantifications both for static and dynamic ^2^H labeling analysis is the conversion of the measured resonances into concentrations. The properties of the ^2^H spectrum are advantageous for spectral quantification, with the possibility to use the natural abundance signal (0.0156%) from HDO in the metabolite spectrum acquired without water suppression. Considering the very low natural abundance of ^2^H, the presence of D_2_O can be neglected (2.43*10^–8^ of all water molecules). This is a major advantage compared to other nuclei used for tracer experiments, such as ^13^C, which typically lacks internal references for concentration quantification. Considering the natural abundance of ^2^H, a water brain tissue fraction of 0.8, a water concentration of 55 M and the 2 hydrogens in a water molecule that can be labeled, the natural water ^2^H concentration is given by [17]: 55 × 2 × 0.0156% × 0.8 = 13.7 mM. With the sparsity of the ^2^H spectra and the good-quality baseline, spectral fitting was achieved with various approaches, such as Matlab home-made peak fitting [16, 17, 32, 86], jMRUI/AMARES [87], or LCModel [88]. Further advantages of using HDO as internal reference are that voxel-wise quantification can correct for various local SNR related to specific *B*_1_ profiles of the RF coil used. Water content maps have also been used to further correct for local water concentration variations. For fast MRSI acquisition strategies using short *T*_R_, *T*_1_ corrections need to be applied [32, 36]. Further improvements have been made by applying voxel-wise water fraction corrections, taking into account tissue water fraction inputs measured from MPRAGE acquisitions, assuming water fractions of 0.71, 0.83 and 1.0 for WM, GM and CSF [74].

It is interesting as highlighted by De Feyter and de Graaf [25] that the natural abundance of ^2^H does not represent an absolute constant but exhibits geographic variability and reports have used values typically ranging from 0.0115% [16] to 0.0156% [17], depending whether the value is based on standard mean oceanic water (0.0156%) [81] or fresh water in temperate climates (0.0115%) [89]. This represents about 25% difference, which is substantial and should be considered with care, in particular when comparing studies from different groups. On the other hand, GC–MS or NMR measurements of ^2^H content in body fluids [90] could provide a reference for in vivo HDO basal concentration. Moreover, the metabolic endpoint of ^2^H is water, which will therefore increase HDO concentration over an infusion experiment. This was already measured in pioneering ^2^H MR labeling studies [17], and initial water ^2^H signal before infusion is therefore typically used as concentration reference. This will consequently also limit the repeatability of such an experiment since ^2^H wash-out from the body might take some time. Reproducibility through repeated scans in humans showed some quantification biases attributed to incomplete clearance of ^2^H from the previous labeling experiment, up to 45 days later [74]. This could be a potential limitation compared for example to FDG-PET experiments, where no significant label is left 24 h after the tracer infusion (^18^F half-life = 110 min). However, ionizing radiation dosimetry considerations limit the repeatability of PET experiments.

### Metabolic modeling

Initial milestone results have clearly shown the potential of ^2^H MR to enable metabolic turnover tracking [17] following ^2^H-Glc infusion or to use the inherent magnetic properties of ^2^H, in particular its short *T*_1_, enabling fast spatial phase encoding with short *T*_R_, which showed interesting post-infusion steady-state maps of Glc, water, Glx, and Lac in pathological conditions such as in brain tumors [16] with ^2^H-Glc or ^2^H-Ace infusion. For metabolic flux mapping with dynamic ^2^H MRSI, the combination of these two approaches has to be used, finding the best compromise between temporal and spatial resolution with sufficient SNR to quantify reliably the labeled compound and sufficient temporal resolution to characterize the isotopic turnover of the observed metabolites. Typically, a target of at least 10 min temporal resolution and an infusion duration of 60 to 120 min are necessary for a stable characterization of isotopic turnover from glucose or acetate metabolism [82].

### Kinetic information

**Fig. 10:**
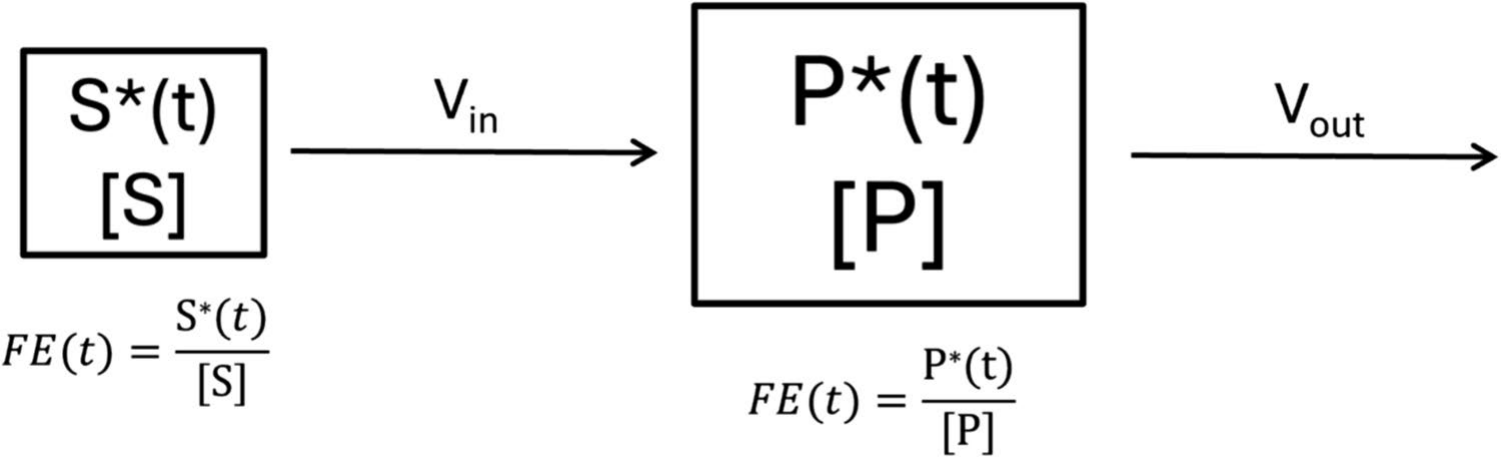
Example of a single product metabolic pool *P* with an influx *V*_in_ from a substrate *S*. S*(*t*) and P*(*t*) represent the time-varying labeled concentrations, while [*S*] and [*P*] are the pool sizes, typically assumed at metabolic steady-state. FEs are the corresponding fractional enrichments. At metabolic steady-state, i.e., with only labeling turnover effects and no mass effects, *V*_in_ = *V*_out_

When analyzing the labeling turnover of X-nuclei, such as ^13^C and ^2^H, compartmental metabolic modeling methods typically assume metabolic steady-state (i.e., no change in metabolites total concentrations) and constant metabolic fluxes over the duration of the experiment. The labeling kinetics of a given metabolic pool (Fig. 10) is then typically given by the following labeling equation [91]:

$$\frac{{{\mathrm{d}}P^{*} (t)}}{{{\mathrm{d}}t}} = \frac{{S^{*} (t)}}{[S]}V_{{{\mathrm{in}}}} - \frac{{P^{*} (t)}}{[P]}V_{{{\mathrm{out}}}} .$$

For a product pool *P* with total concentration [*P*], labeled concentration P*, getting label from the substrate S* with total concentration [*S*] through an inflow *V*_in_ and losing label through *V*_out_. For a simple two-pool system like this, as illustration, the general solution is given by:

$$P^{*} (t) = \int\limits_{0}^{t} {V_{{{\mathrm{in}}}} \frac{{S^{*} (t)}}{[S]}} \exp \left( {\frac{{V_{{{\mathrm{out}}}} }}{[P]}\left( {t^{\prime} - t} \right)} \right){\mathrm{d}}t^{\prime}.$$

This equation shows that two other measurements are critical to estimate metabolic fluxes from metabolites turnover curves. First, the time evolution of the substrate fractional enrichment (S*(*t*)/[*S*]), known as input function of the metabolic system, drives the label into the product pool through *V*_in_. A well-characterized input function is therefore critical to derive the model turnover curves. Second, the pool size of the product pool [*P*] directly enters the typical turnover time of the considered product in the ratio *V*_out_/[*P*]. This reflects the fact that to extract a metabolic flux in micromole/g/min from a turnover rate constant measured from the labeled P*(*t*) time course, the size of the metabolic pool [*P*] also needs to be known. A same turnover time would be reached for a constant ratio *V*_out_/[*P*]. Conversely, a larger labeling signal could reflect a larger pool size [*P*] and not necessarily a larger flux rate [82]. As a consequence, if not measured, the assumed pool sizes of the measured products should be chosen wisely as they directly impact the derived metabolic fluxes.

### ^2^H metabolic modeling studies

Lu et al. [17] have exploited the high temporal resolution of un-localized ^2^H MRS to model ^2^H Glx and Glc turnover data and determine CMR_glc_ and *V*_TCA_ concurrently in rats at 16.4 T, in a coil-localization acquisition approach (pulse-acquire un-localized experiment), using a fitted blood arterial glucose level and enrichment as input function, following a brief (2 min) intravenous infusion of d-glucose-6,6-d_2_. Although partially including non-cerebral tissue due to the coil localization approach, the authors derived metabolic fluxes for CMR_glc_ and *V*_TCA_ in agreement with previously reported values in dynamic ^13^C MRS experiments. This first metabolic flux interpretation of ^2^H dynamic data was directly based on the ^13^C metabolic approach developed in the previous 2 decades [92–94]. The measurement of a single labeled position in Glx as metabolic accessible kinetic pool, as compared to the multiple carbon positions of separate Glu, Gln, and Asp accessible with ^13^C limited the accessible metabolic information, facing the risk of model under-determination. Hence, the model needed fix parameters inputs, such as the pool sizes [Glc]_brain_, [Lac], [Glx], and [Gly], the Glc transporter parameter *K*_T_, and the lactate efflux *V*_out_. Although maximizing the accessible chemical information for SNR and temporal resolution with no spatial encoding, only low ^2^H Lac labeling could be identified and its kinetics was not fitted with the metabolic model. As also shown in this publication in which accumulation of labeled water was clearly demonstrated, HDO and its increase through metabolism can be detected with high SNR. However, this metabolic labeling of water does not reflect local metabolism but rather body metabolism through HDO that washed into the region of interest from other tissues in the body and is therefore difficult to interpret.

A similar metabolic modeling approach was used by Kreis et al. [36] to evaluate the use of ^2^H MRSI for quantitative mapping of tumor glycolytic flux and to assess response to chemotherapy. Besides steady-state Lac-to-Glc and Lac-to-water ratios, this study showed deeper insights with dynamic interpretation of the ^2^H concentrations. The metabolic model applied here was also derived from previous ^13^C metabolic modeling approaches. This study focused on the measurable Lac ^2^H turnover in a murine tumor model and measured maps of glycolytic flux *V*_max_ from glucose to lactate, typical of Warburg effect metabolism in tumors, using a metabolic model with 6 free parameters. Some additional hypothesis needed to be done for the pool size estimate of brain extracellular Glc, and the Lac pool size was indirectly adjusted through the efflux rate constant. In that particular metabolic situation, ^2^H Lac was measurable but not Glx. Hence, the metabolic model depicted the second branch of glucose utilization through non-oxidative metabolism.

Li et al. [74] revisited their metabolic modeling approach for whole-brain quantitative imaging of glucose consumption (CMR_glc_), lactate production (CMR_lac_), and tricarboxylic acid (TCA) cycle (*V*_TCA_) in human at 7 T with 0.7 cc nominal voxel size and 2.5 min/image. Lac could be measured after the application of the SPICE de-noising scheme. They developed a quantification method correcting for magnetization saturation factors (SFs), related to *T*_1_ partial saturation at short *T*_R_, and brain tissue water fraction. It also enabled through regression the measurement of “pure GM” and “pure WM” ^2^H Glx turnover. The metabolic model comprised 4 adjusted parameters CMR_glc_, CMR_lac_, *V*_TCA_, and *T*_max_, while the pool sizes and further the metabolic fluxes or the parameters were fixed (lactate *V*_out_, *K*_T_ = 15 mM/min, *V*_x_ = 30 mM/min).

SPICE de-noising enabled higher SNR and spatial resolution, but the impact of the temporal “smoothing” induced by SPICE on the metabolic flux derivation remains to be evaluated.

### Label loss

Compared to ^13^C labeling experiments, dynamic ^2^H MRSI studies are, however, harder to interpret from a biochemical point of view. The limited chemical shift dispersion typically leads to the measurement of a single labeling position, typically Glx C4 from [6′,6]-^2^H glucose metabolism, as compared to ^13^C which enables the measurements several metabolites carbon positions, such as Glu C4,C3, and C2, in consecutive TCA cycles [82, 83, 85], and even further multiply labeled metabolites with fine structure ^13^C–^13^C coupling patterns [95, 96]. This is linked to the fact that the ^2^H atoms from Glx C4 are lost to water during the following turn of the TCA cycle [25]. Although the loss of isotopic labels in the investigated biochemical pathways generally reduces the scope of accessible information, the absence of labeling at positions C3 and C2 is advantageous in this context. It avoids the occurrence of complex labeling patterns within the ^2^H spectrum, which is characterized by limited chemical shift dispersion and inherently broad resonances.

Besides the lower biochemical information content of ^2^H spectra, an important point to consider for the quantification of metabolic reactions with ^2^H as compared to ^13^C are kinetic isotope effects and label losses or isotope exchanges with water. de Graaf et al. [97] showed in rats tissue extracts and glioma cells that kinetic isotopes effects were small for ^2^H, both when using [6,6-^2^H_2_]-glucose and [2-^2^H_3_]-acetate as metabolic substrate (4–6%). On the other hand, substantial label loss toward glutamate, glutamine, and lactate were measured (15.7 to 41.5% ^2^H signal loss for [6,6-^2^H_2_]-glucose). The measurement of these substrate- and pathway-specific label losses and their incorporation in metabolic modeling are therefore key for quantitative studies aimed at mapping metabolic fluxes in vivo using ^2^H-labeled substrates with ^2^H MRSI.

## Conclusion and future outlook

Deuterium metabolic imaging has progressed from an experimental technique to a flexible platform for in vivo metabolic mapping, with a broad freedom in tracer choice and acquisition design. Advances in RF hardware, spatial–spectral non-Cartesian sampling, bSSFP-based DMI, and interleaved multinuclear approaches have markedly improved SNR efficiency and spatiotemporal resolution at clinical and ultra-high fields. In parallel, indirect ^1^H-based detection and model-based, low-rank or AI-driven reconstructions are beginning to reduce scan time and improve robustness, positioning DMI as a powerful, non-ionizing complement to FDG-PET and ^13^C-based methods.

As 3 T MR systems represent the dominant clinical platform, the recent translation of DMI from ultra-high to clinical field strengths represents a key milestone toward wider clinical translation. Further progress will depend on a broad vendor support for multinuclear implementations along with standardized RF coil solutions and streamlined reconstruction and quantification pipelines that are robust in routine settings. At 3 T, the reduced chemical shift dispersion and the inherently lower sensitivity can limit fitting robustness and achievable spatial resolution, making improvements in SNR efficiency essential. Harmonized protocols, SNR efficient acquisitions such as bSSFP-based approaches, dedicated multi-channel RF coils, and automated de-noising will be critical to establish clinically acceptable spatial resolution within feasible scan times.

In this context, integrating deep learning-based algorithms into DMI pipelines, e.g., for spectral de-noising, accelerated reconstruction and automated spectral fitting could further facilitate clinical adoption at 3 T. Particularly, fast and automated fitting could be integrated directly on the scanner to enable near real-time visualization of metabolic maps immediately after the acquisition, supporting workflow integration and prospective quality assessment.

In addition, accelerated DMI, multinuclear interleaving, and QELT-based ^2^H detection offer a realistic path to integrating metabolic imaging into routine workflows. As these barriers are addressed, DMI is poised to evolve from single-center feasibility studies to multi-center trials and, ultimately, a clinically relevant tool for characterizing metabolism and monitoring therapy across a range of neurological, oncological and cardio-metabolic diseases.

## Data Availability

There are no data generated to be shared.
